# Improving OCT Image Segmentation of Retinal Layers by Utilizing a Machine Learning Based Multistage System of Stacked Multiscale Encoders and Decoders

**DOI:** 10.3390/bioengineering10101177

**Published:** 2023-10-10

**Authors:** Arunodhayan Sampath Kumar, Tobias Schlosser, Holger Langner, Marc Ritter, Danny Kowerko

**Affiliations:** 1 Junior Professorship of Media Computing, Chemnitz University of Technology, 09107 Chemnitz, Germany; arunodhayan.sampath-kumar@cs.tu-chemnitz.de (A.S.K.); tobias.schlosser@cs.tu-chemnitz.de (T.S.); 2 Professorship of Media Informatics, University of Applied Sciences Mittweida, 09648 Mittweida, Germany; langner@hs-mittweida.de (H.L.); ritter@hs-mittweida.de (M.R.)

**Keywords:** ophthalmology, ophthalmology diseases, OCT biomarkers, OCT segmentation, computer vision and pattern recognition, machine learning, deep learning

## Abstract

Optical coherence tomography (OCT)-based retinal imagery is often utilized to determine influential factors in patient progression and treatment, for which the retinal layers of the human eye are investigated to assess a patient’s health status and eyesight. In this contribution, we propose a machine learning (ML)-based multistage system of stacked multiscale encoders and decoders for the image segmentation of OCT imagery of the retinal layers to enable the following evaluation regarding the physiological and pathological states. Our proposed system’s results highlight its benefits compared to currently investigated approaches by combining commonly deployed methods from deep learning (DL) while utilizing deep neural networks (DNN). We conclude that by stacking multiple multiscale encoders and decoders, improved scores for the image segmentation task can be achieved. Our retinal-layer-based segmentation results in a final segmentation performance of up to 82.25±0.74% for the Sørensen–Dice coefficient, outperforming the current best single-stage model by 1.55% with a score of 80.70±0.20%, given the evaluated peripapillary OCT data set. Additionally, we provide results on the data sets Duke SD-OCT, Heidelberg, and UMN to illustrate our model’s performance on especially noisy data sets.

## 1. Introduction and Motivation

Humans are highly dependent on their vision for social interactions. Globally, 39 million people are visually impaired and are aged above 50 years [1]. The primary causes of blindness are cataracts (51%), glaucoma (8%), age-related macula degeneration (AMD) (5%), corneal opacities (4%), uncorrected refractive errors and trachoma (each 3%), and diabetic retinopathy (1%) [2,3]. In the KORA-Age study conducted in the southern part of Germany, 822 participants (49.6% women, 50.4% men, aged 68–96 years) were asked standard questions related to eye diseases. The most common eye diseases were cataracts (36%), dry eyes (15%), glaucoma (9%), and AMD (8%) [4]. Most of the participants were suffering from glaucoma or AMD while having cataracts. Another study estimated the incidences of severe visual impairment and blindness by using Germany’s largest state blind registry and their projection rates for Germany in 2010 and 2030. The major causes of blindness and visual impairment were AMD (50%), glaucoma (15%), and diabetic retinopathy (10%) [5].

A non-invasive three-dimensional imaging modality used in eye clinics for the diagnosis of pathologies is optical coherence tomography (OCT) [6], a non-invasive imaging technique that uses light waves to capture biological tissue in high resolution. It is an important technique in organs where the traditional microscopic tissue diagnosis method employing a biopsy is unavailable [7]. For OCT, near-infrared light with wavelengths ranging from about 800 to 1300 nm is typically employed, enabling deeper penetration into tissues such as the retina of the human eye compared to visible light [8]. Here, the OCT B-scan provides a two-dimensional, cross-sectional view to allow the visualization of the retina and cornea, which aids in the diagnosis of diseases such as AMD, glaucoma, and diabetic retinopathy [9].

Medical image semantic segmentations are a wide area of research that involves the extraction of regions of interest (ROIs) from medical images, such as in the form of computed tomography (CT) scans, magnetic resonance imaging (MRI) scans, X-ray scans, and OCT scans [10]. However, manual segmentation is an incredibly time-consuming task [11]. Therefore, current approaches also encompass the development of algorithms incorporating machine learning (ML) and deep learning (DL) in the form of deep neural networks (DNN), whereas models trained with OCT imagery enable the (semi-)automated classification of OCT biomarkers [12] and the segmentation of OCT imagery into the different layers of the human eye (retinal layers) [13,14]. Finally, (semi-)automated recommender systems can assist doctors with the identification and location of abnormalities such as AMD [15], diabetic retinopathy [16], and glaucoma [17], ultimately aiding in the reduction of visual impairments and blindness among patients.

### 1.1. Related Work

The success of DNNs such as convolution neural networks (CNN) in object detection has helped researchers to explore the feature learning capabilities of prediction problems such as image segmentation [18,19,20]. Research on semantic pixel-wise segmentation is an ongoing topic that is driven by complex data sets. Prior to the development of DNNs, the most effective techniques mainly used manually created characteristics to categorize individual pixels [21]. To estimate the class probabilities of a ROI, a patch is typically fed through a machine learning classifier, such as a random forest classifier [22], a voting classifier [23], or boosting [24]. Recent medical image segmentation methods primarily utilized autoencoder-based DNNs for end-to-end segmentation [25,26]. The most commonly used OCT-based segmentation models are the fully convolutional networks (FCNs) [27] and U-Net [28]. Other segmentation methods applied to medical image segmentation are the region-based CNNs (R-CNN) [29] and instance segmentation [30].

In terms of the state-of-the-art models for macular-based OCT segmentation, ReLayNet was proposed for the semantic segmentation of macular OCT B-scans into their retinal layers and fluid masses [25]. This method paved the way for a baseline for the automatic segmentation of retinal OCT layers. An attention-guided channel-to-pixel CNN for retinal layer segmentation with choroidal neovascularization was designed by [17], where a channel-to-pixel block is utilized along with an edge loss function to segment the retinal layers with blurry boundaries. The attention mechanism has been employed to address the sizeable morphological variation among retinal layers [31]. The work of [32] utilized a multiscale and dual attention (MDAN) U-Net with multiscale features and attention mechanisms to further improve the segmentation performance of U-Net. Additionally, commonly used OCT segmentation methods are the feature pyramid networks (FPNs) [33] for global feature extraction, followed by a Gaussian process and feature alignment with epistemic uncertainty [34,35]. Subsequently, [36] developed a so-called fully convolutional instance segmentation (FCIS) network with a segment proposal network and an object detection system.

Recent natural language processing (NLP) developments using recurrent neural networks (RNNs) have been explored for OCT segmentation. These models consider sequences between different scans for processing pixel sequences [37,38]. The work of [39] developed a polyp detection system using an autoencoder network with an encoder (pre-trained VGG19) and a decoder (U-Net) [28] branch to segment colon cancer cells. Y-Net [40], a model inspired by U-Net, was developed to segment cancer cells irrespective of shape, size, texture, and orientation. With state-of-the-art autoencoders, the encoder often produces low-resolution representations, while the decoder is responsible for producing multidimensional features for each pixel for classification [41]. The authors utilized a single-stage training process where the encoder was the VGG16 with weights pre-trained on ImageNet. The decoder was an FCN architecture that learns to upsample its input feature maps along the encoder’s feature map to produce the input to its respective decoder [40].

Often, decoder networks progressively add existing networks as single-stage approaches until no further improvement is achieved [42]. Further, prediction capabilities can be improved by appending an RNN to the existing FCN network as RNN layers mimic conditional random fields’ (CRF) sharp boundary delineation capabilities while exploring the feature representation of the FCN [30]. Recently developed segmentation architectures using DNNs are not fed forward during the inference time [43,44]. They require aids such as region proposal networks for inference. Multiscale deep architectures have become a common approach for feature extraction. They use input images at multiple scales with their corresponding feature maps from different layers [45]. Others employ a combination of feature maps from different layers in a single deep architecture [46]. However, the common idea of incorporating global and local contexts is to extract features at multiple scales [47].

For peripapillary retinal images, ref. [48] developed an automatic segmentation method. With their approach, the boundaries of the optic discs (left and right) were determined based on the estimation of the position of Bruch’s membrane openings in radially OCT B-scans by combining a CNN with a multigraph search algorithm that supports the segmentation of retinal boundaries. Finally, a Dilated-Residual U-Net (DRUNET) was proposed by [49] to segment five retinal layers and optic discs that are not thoroughly segmented from their connecting tissues.

In comparison to approaches that are commonly deployed for image segmentation, generative approaches such as variational autoencoders (VAE) [50] and generative adversarial networks (GAN) [51] are currently being investigated regarding their diagnostic abilities within ophthalmology [52]. In [52], the authors report on the capabilities of GANs, ranging from the segmentation of images to their augmentation, denoising, domain transfer, super resolution, post-intervention prediction, and feature extraction. However, given their limitations, such as the risk of generating additional noises and artifacts, generative approaches to OCT image segmentation are still being developed, whereas AUC scores within the range of 92 to 97% are being reported [52,53].

### 1.2. Contribution of This Work and Future Prospects

In this contribution, we propose a machine-learning-based multistage system of stacked multiscale encoders and decoders to perform the retinal layer segmentation task. This approach is motivated by current developments by [17] on the segmentation of peripapillary OCT imagery, where the segmentation task is realized using a multiscale graph convolutional network (GCN)-assisted two-stage network for the OCT image segmentation task. Here, we extend this approach by utilizing an approach where multiple encoders and decoders are stacked to obtain improved segmentation scores. As the results of this segmentation task can be potentially leveraged for the following classification stage, the importance of the segmentation task itself is emphasized, given the retinal layers’ physiological and pathological states.

In regards to future prospects, retinal layer segmentation could serve as a visual aid during the education of (medical) students as well as medical assistants working towards becoming professionals in ophthalmology. With the provided retinal layer segmentations, they are further enabled to learn, explore, and understand the exact locations as well as the extent of the retinal conditions, which is crucial for effective treatment planning. In particular, retinal disorders and pathological conditions emerging from fluids are accessible in a quantitative manner. Combining multiple OCT B-scan segmentations could even enable future three-dimensional visualizations, therefore providing a better understanding of the anatomical structures and pathological conditions. Additionally, the obtained segmentations can be further utilized for subsequent processing steps such as OCT biomarker classification, visual acuity predictions, as well as treatment predictions and adjustments. By leveraging community data sets with different physiological and pathological states, the diagnostic skills of healthcare professionals can be supported, and even patients can be educated about their eye conditions.

### 1.3. Section Overview

In the following text, our contribution is structured as follows: Firstly, our materials, methods, implementation of our data sets, model architecture, training setup, loss functions, and evaluation principles are introduced in Section 2. Our test results, evaluation, and discussion include a quantitative evaluation of the OCT image segmentation for retinal layers by utilizing our proposed system in comparison to currently developed and investigated baseline approaches in Section 3. In addition to our quantitative results, a qualitative evaluation using segmentation visualizations is provided to illustrate our results. Finally, we provide conclusions on the benefits of our system while giving additional insights into future developments and further possibilities for improvement.

## 2. Materials, Methods, and Implementation

First, a suitable data corpus has to be defined to realize the OCT image segmentation of retinal layers. For this purpose, Section 2.1 introduces our data sets with their OCT imagery and segmentations. The model architecture used for our machine-learning-based multistage system of stacked multiscale encoders and decoders is introduced in Section 2.2 with its training setup and utilized loss functions presented in Section 2.3 and Section 2.4. Finally, our evaluation principles and used metrics are highlighted in Section 2.5.

### 2.1. Data Sets

**Figure 1:**
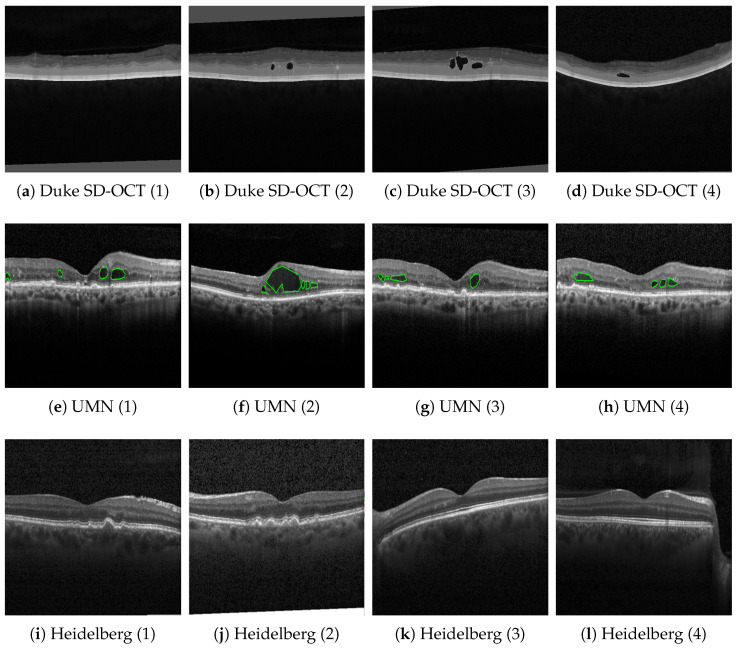
Overview of our public data sets for qualitative assessment, Duke SD-OCT [14] (**top row**), UMN [54] (**middle row**), and Heidelberg [55] (**bottom row**), with and their annotations, if available. For the Duke SD-OCT data set, the retinal layers are highlighted in terms of different levels of brightness. For the UMN data set, areas with fluids are additionally available (highlighted in green). For the Heidelberg data set, no annotations are available.

In the following text, the data sets used for the quantitative as well as the qualitative evaluation are introduced. For this purpose, Figure 1 gives an overview of our public data sets that were used for the qualitative assessment, Duke SD-OCT [14] (top row), UMN [54] (middle row), and Heidelberg [55] (bottom row), with and their annotations, if available. For the Duke SD-OCT data set, the retinal layers are highlighted in terms of their different levels of brightness. For the UMN data set, areas with fluids are additionally available. For the Heidelberg data set, no annotations are available. Additionally, Table 1 gives a tabular overview of the public data sets used in our further experiments.

#### 2.1.1. Peripapillary Data Set

**Figure 2:**
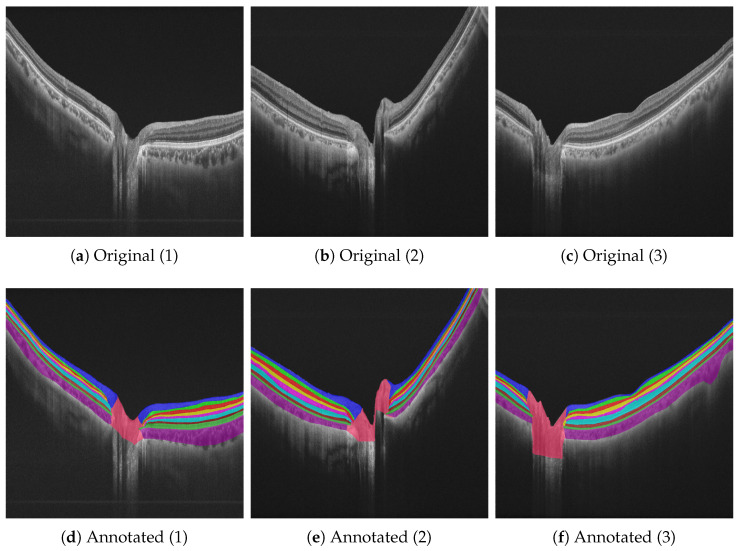
Overview of our data set with two selected samples and their visualized retinal layers following the color scheme presented in Table 2 as a legend for the present visualizations. Table 2 gives an overview of our data set with its samples and the proposed data set split ratio. In the top row, exemplary peripapillary OCT images are shown with their annotations in the bottom row.

The peripapillary OCT data set [17] used for the evaluation of our system comprises 122 randomly selected radial OCT B-scans from 61 patients with 10 manually annotated labels (also see Figure 2). For the original data set, two graders annotated the OCT B-scans via ITK-SNAP [56] with the help of glaucoma specialists. The obtained annotations reflect the consensus of all graders [17]. With the provided radial OCT B-scans, 12 image slices are available per OCT B-Scan, where for each patient, one eye was imaged. Each patient contributed two randomly selected image slices, resulting in 122 image slices overall. A horizontal flip augmentation, additive Gaussian noises, and contrast adjustment were performed on the training data set to augment the number of samples by a factor of two, resulting in 244 samples in total. Here, we follow this principle to allow a comparison of the results previously obtained by [17]. Table 2 shows the utilized OCT image segmentation data set with its retinal layers, abbreviations, and color scheme for visualization [17]. Our training, validation, and testing sets were split with a data set split ratio of 60/20/20, resulting in 148/48/48 images, respectively. The image resolution of 1024×992 pixels is constant over all images and data subsets. All images contain annotations of layer segmentations, as given in Table 2.

#### 2.1.2. Duke SD-OCT Data Set

The Duke University provides the publicly available Duke SD-OCT data set [14], which comprises 110 OCT B-scans recorded from 10 diabetic macular edema (DME) patients. The data set includes fluid and non-fluid manual annotations of eight boundaries by two ophthalmologists, encompassing RNFL, GCL, INL, OPL, ONL, the inner segment ellipsoid (ISE), and the outer segment retinal pigment epithelium (OS-RPE).

#### 2.1.3. UMN Data Set

The Minnesota Ophthalmology University Clinic collected the UWN data set [54], which comprises 600 OCT B-scans from 24 AMD patients. Within the UMN data set, retinal fluid regions—intraretinal fluid (IRF), subretinal fluid (SRF), and pigment epithelial detachment (PED)—were manually annotated and assessed by two ophthalmologists.

#### 2.1.4. Heidelberg Data Set

The Heidelberg data set [55] comprises 108,312 OCT B-scans recorded from 4686 patients with retinal fluid annotations, including 37,206 images with choroidal neovascularization (CNV), 11,349 images with DME, 8617 images with drusen, and 51,140 healthy images. The retinal fluid annotations were manually annotated with a tiered grading system. The employed undergraduate and medical students were first-tier graders who reviewed the diagnostic information and discarded OCTs contaminated by severe artifacts. The following four ophthalmologists were second-tier graders who independently graded the images as CNV, DME, and drusen.

### 2.2. Model Architecture

**Figure 3:**
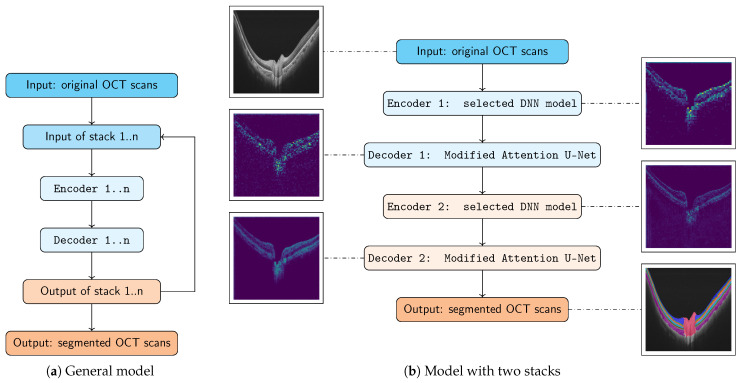
The model architecture and framework of our proposed model. (**a**) The general model used to stack multiple encoders and decoders is illustrated. (**b**) The model with two stacks of encoders and decoders is shown. For visualization purposes, cyan and orange represent the input and the output, light cyan represents the first stack with encoder 1 and decoder 1, and light orange represents the second stack with encoder 2 and decoder 2.

The model architecture of our proposed system where multiple encoders and decoders are combined as stacks is shown in Figure 3. In Figure 3a, the general model is illustrated. Figure 3b shows our proposed and implemented architecture, consisting of two main parts: stack 1 with encoder 1 and decoder 1 (Section 2.2.1), where the first decoder, our Modified Attention U-Net (Section 2.2.3), uses a local context, as well as stack 2 with encoder 2 and decoder 2 (Section 2.2.2), where the second decoder, again our Modified Attention U-Net, uses a larger context as the denoiser. The proposed segmentation network predicts the segmentation map given an input OCT scan and its corresponding labels. A detailed description of the realized model and its first stack, its second stack, and our Modified Attention U-Net as decoders 1 and 2 is provided in the following text.

#### 2.2.1. Stack 1: Encoder 1 + Decoder 1 (Local Context)

Within the first stack, the encoder captures contextual pieces of information of the input data in high resolution to show lower-dimensional representations. Each encoder layer possesses spatial attention, whereby the network can focus on certain areas of a feature map. The decoder operates at a higher resolution with a focus on fine-grained details. The term “local context” implies that this part of the architecture looks at smaller, more localized patterns or features in the input, for which the local context is essential for capturing details and subtle nuances in the data.

#### 2.2.2. Stack 2: Encoder 2 + Decoder 2 (Denoiser)

Within the second stack, the architecture can naturally introduce multiscale processing by utilizing a second encoder. The second encoder allows the network to refine and further process the features from the output of the first decoder. By doing so, the model can capture higher-order interactions and details that might not be captured in the first stack. The features or outputs of decoder 1 possibly show inconsistencies, inaccuracies, or noise, and encoder 2 can be trained to correct or filter these artifacts by focusing on more robust features. The second decoder’s primary purpose is to refine or “clean” the output. The decoder looks at broader patterns and features within the data. Subsequently, a larger context allows the model to understand the overall structure and global patterns, which can help to refine or denoise the output. Denoising models remove noise or unwanted artifacts from the data. In images, this could lead to the reduction of pixel-level artifacts or the smoothing out of regions.

#### 2.2.3. Modified Attention U-Net

There are two significant advantages to the addition of an attention mechanism before the upsampling step. Firstly, there is focused information upsampling. The attention mechanism enhances certain features and suppresses less important ones. Hence, during upsampling, only the most important information gets propagated to a higher resolution, leading to potentially more accurate reconstructions. Secondly, there is contextual awareness. The attention mechanism inherently takes a larger contextual view of the input feature map. This broad contextual awareness can guide the upsampling process by applying it before upsampling. This means that the resultant higher-resolution feature maps can be more contextually aligned with the broader features of the image data, potentially leading to better global coherence in the output.

The general architecture of U-Net [28] is symmetric and comprises two major parts. The left part is called the contracting part, which consists of the convolution process. The right part is the expansive part with transposed two-dimensional convolutional layers (upsampling). Motivated by vision transformers [57], we introduce an attention mechanism with residual learning [58,59] to facilitate advanced information fusion between feature maps in our network architecture. This module is crafted to integrate two feature maps: the primary feature map *x* and a secondary or skip feature map *skip*. We specifically employ concatenation as our fusion method to optimize the information flow. Given a primary feature map *x* with a channel size of $C_{x}$ as well as a skip feature map *skip* with a channel size of $C_{\mathrm{skip}}$, integration using concatenation is formalized in Equation (1). The primary and skip connection feature maps are concatenated along the channel dimension, resulting in a channel size of $C_{x}+C_{\mathrm{skip}}$. Subsequently, a 1×1 convolution is employed to transform this merged feature map back to a channel size of $C_{x}$. Concatenation-based fusion is designed to seamlessly integrate contextual information from the skip connection, enhancing the expressive power of our network.

(1) $$x^{\prime }={\mathrm{Convolution}}_{1\times 1}\left(C_{x}+C_{\mathrm{skip}}\to C_{x}\right)\left(\left[x,\mathrm{skip}\right]\right)$$

For implementation purposes, we used the Segmentation Models PyTorch library (Segmentation Models PyTorch library, https://smp.readthedocs.io/en/latest/index.html, accessed on 4 October 2023) [60] for U-Net while modifying its functionality by adapting it with the aforementioned attention mechanism.

### 2.3. Training Setup

Our training setup included the Adam optimizer [61] with a concatenating cosine annealing linear scheduler with an initial learning rate of 0.001, decaying by a factor of 0.01×learningrate, and a batch size of 32. For validation, we used four-fold cross-validation for our main experiments. Our models were trained for 75 epochs, whereas early stopping was introduced to prevent overfitting when no further training or validation progress could be observed within the earlier stages of the training process. As a hyperparameter tuning strategy, we utilized a random search for the selection of random combinations of hyperparameter values from pre-defined sets to evaluate our models’ performance levels. This included our optimizer, its learning rate, and its batch size in order to finally obtain the selected model training parameterization. For example, for our batch size, we evaluated batch sizes of 16, 32, and 64. To fine-tune our encoder models, we additionally deployed ImageNet-based weights for the pre-training of the first encoder of the first stack.

To enable future on-site deployment of our realized system as a (semi-)automated recommender system for the OCT image segmentation of retinal layers, we evaluated our setup using general-purpose graphics processing units (GPGPU) within all of the following experiments. Our test environment was solely composed of current consumer-grade hardware. This test environment encompassed (i) our CPU, >>Intel(R) Core(TM) i9-9900K CPU @ 3.60 GHz<< with 7200 BogoMips and a maximum CPU load of 99%, (ii) our GPU, >>TITAN RTX<< with a maximum GPU load of 99%, (iii) our working memory with 128 GB of RAM, as well as (iv) our hard drive (SSD), >>Samsung 970 EVO Plus SSD<< with 500 GB [62].

### 2.4. Loss Functions

Combining different loss functions helps to capture the characteristics of the data better than using a single loss function. For all of our experiments, we computed a combined loss function composed of three popular loss functions, the Sørensen–Dice loss [63], also known as the Dice score, for the evaluation of image segmentations, the Lovász loss [64], and the Tversky loss [65]. For their use, we utilized the Segmentation Models PyTorch library [60], whereas the optimal weights for each loss function were determined via hyperparameter tuning.

#### 2.4.1. Dice Loss

The binary Dice loss function $D_{\mathrm{binary}\mathrm{loss}}$, which is often used for binary segmentation tasks, is based on the binary Dice score $D_{\mathrm{binary}\mathrm{score}}$ [63]. It is defined given the ground truth and the predicted segmentations *X* and *Y*.

(2) $$D_{\mathrm{binary}\mathrm{score}}=\frac{2|X\cap Y|}{\left|X|+|Y\right|}$$

(3) $$D_{\mathrm{binary}\mathrm{loss}}=1-D_{\mathrm{binary}\mathrm{score}}\left(X,Y\right)$$

For multiclass segmentations with *C* classes [66], the Dice loss ${\mathrm{Dice}}_{\mathrm{multiclass}\mathrm{loss}}$ is based on the multiclass Dice score $D_{\mathrm{multiclass}\mathrm{score}}$ with $X_{c}$ and $Y_{c}$ being the ground truth and the predicted segmentations for class *c*.

(4) $$D_{\mathrm{multiclass}\mathrm{score}}=\frac{1}{C}\sum_{c=1}^{C}D_{\mathrm{binary}\mathrm{score}}\left(X_{c},Y_{c}\right)$$

(5) $$D_{\mathrm{multiclass}\mathrm{loss}}=\frac{1}{C}\sum_{c=1}^{C}\left[1-D_{\mathrm{binary}\mathrm{score}}\left(X_{c},Y_{c}\right)\right]$$

#### 2.4.2. Lovász Loss

The Lovász–Softmax loss L [64] is a differentiable surrogate of the intersection over union (IoU) measure. It is defined as follows:

(6) $$L=\sum_{i=1}^{n}\alpha \left(i\right){\left[m\left(i\right)-\phi \left(i\right)\right]}_{+}$$

where:

ϕ(i) = predicted probability sorted in decreasing order
m(i) = the corresponding ground truth label
$p_{i}$ = the cumulative sum of ground truth labels up to index *i*
α(i) = $p_{i}-p_{i-1}$
${\left[x\right]}_{+}$ = denotes the positive part of *x*, defined as max(x,0).

#### 2.4.3. Tversky Loss

The Tversky loss T [65], a generalization of the Dice coefficient, is controlled via α and β influencing the magnitude of penalties for false positives and false negatives, respectively:

(7) $$T=1-\frac{\left|X\cap Y\right|}{\left|X\cap Y|+\alpha |X-Y|+\beta |Y-X\right|}$$

#### 2.4.4. Combined Loss

By combining the Dice loss (Equation (5)), the Lovász loss (Equation (6)), and the Tversky loss functions (Equation (7)), we obtained our combined loss function, which was weighted as follows:

(8) $$\mathrm{Combined}\mathrm{loss}=0.5\times D_{\mathrm{multiclass}\mathrm{loss}}+0.3\times L+0.2\times T$$

### 2.5. Evaluation Principles

For the evaluation, we utilized the Dice score given our obtained OCT image segmentations [67,68] using Equation (4), which, in image segmentation, corresponds to the F1-score used for classification tasks [69,70]. Therefore, the Dice score was used to assess the alignment of our predicted segmentations with their labeled ground truth segmentations.

## 3. Test Results, Evaluation, and Discussion

For the test results, evaluation, and discussion of our machine-learning-based multistage system of stacked multiscale encoders and decoders, the following sections give an overview of our segmentation results using the introduced OCT image segmentation data sets presented in Section 2.1. For this purpose, a differentiation between the quantitative (Section 3.1) and qualitative results (Section 3.2) is made, for which we provide a discussion in the context of currently deployed comparable deep learning models.

### 3.1. Quantitative Results

**Figure 4:**
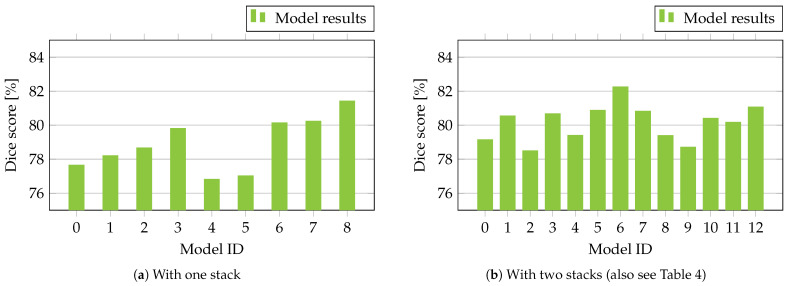
Visualization of the quantitative results with (**a**) one stack and (**b**) two stacks from Table 3 and Table 4.

For the evaluation, we utilized the EfficientNet (B0–B5) [71], ResNet34D and ResNet50D [58,72] models, as well as SEResNeXt50-32x4D [73,74] as encoders, whereas our Modified Attention U-Net was deployed as the decoder (decoders 1 and 2). Our models were pre-trained on ImageNet while utilizing the parameterizations given by their original authors. Table 3 gives an overview of our obtained results with one stack of our proposed system, while Table 4 gives an overview with two stacks using our deployed models. Additionally, all shown results are visualized in Figure 4 with their respective segmentation performances in terms of the Dice score. These models were utilized as they are considered to be well-established baselines for deep neural network performance as they deploy principles such as uniformly scaled network features [71], residual learning [58,59], and attention mechanisms such as squeeze-and-excitation blocks [73] while showing a wide range of applications in different medical image segmentation and classification tasks [75,76,77,78].

In addition, the following baseline deep neural network results are reported. Given one model without the explicit differentiation between encoders and decoders as well as their stacks, models such as U-Net, DRUNET, and ReLayNet allow for out-of-the-box segmentation. The previously reported scores were 80.5±0.4, 80.6±0.4, and 80.4±0.4% for U-Net [28], DRUNET [49], and ReLayNet [25], respectively [17].

Given our results in Table 3, using SEResNeXt50-32x4D and our Modified Attention U-Net as the encoder and decoder led to the best segmentation score when only utilizing one stack. Following this observation, the best segmentation scores were obtained from deploying SEResNeXt50-32x4D and ResNet34D as encoder 1 and encoder 2 with Modified Attention U-Net as decoders 1 and 2. With the worst-performing combination, EfficientNet B4 and EfficientNet B5 as encoder 1 and encoder 2, a range or model-based improvement of about 3.76% was obtained. Given the different obtained results, Table 5 shows a summarized comparison with the current state-of-the-art method given the data set presented by [17] with a single-stage approach. Furthermore, our results with a single encoder and decoder with attention pooling (one stack with SEResNeXt50-32x4D) and our best result with stacked encoders and decoders with attention pooling (two stacks with SEResNeXt50-32x4D and ResNet34D) are depicted. In comparison, our system resulted in an improvement in the segmentation score by 1.55% compared with the single-stage baseline. From the single-stage baseline to one stack, an improvement of 0.72% was obtained, whereas the final improvement by adding stage 2 resulted in an additional improvement of 0.83%.

From our experience, using the same encoder for different stacks leads to increased computational costs while facing problems such as vanishing gradients. Additionally, overfitting may appear in deeper models. To further investigate this observation, we added the three best-performing encoders from Table 4, deploying them as encoder 1 and encoder 2, respectively. These were ResNet34D, ResNet50D, and SEResNeXt50-32x4D. For ResNet34D and ResNet50D, no improvement could be obtained compared to our results with one stack, while SEResNeXt50-32x4D even resulted in a reduced performance in comparison, despite being the best-performing encoder in the three additional test runs.

### 3.2. Qualitative Results

**Figure 5:**
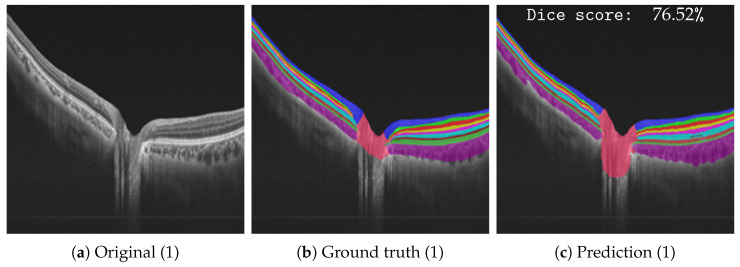

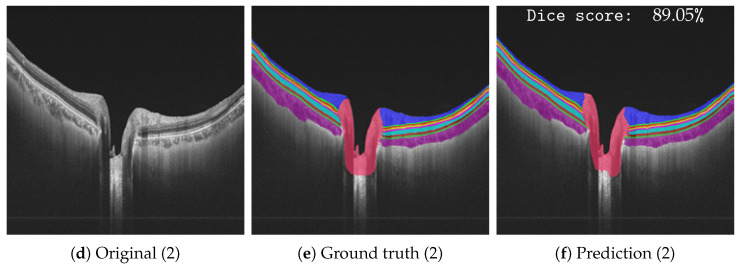
Qualitative peripapillary data set with the original, ground truth segmentation, and segmentation model results as a color-coded overlay. The individual, class-wise Dice scores are shown in Table 6.

**Figure 6:**
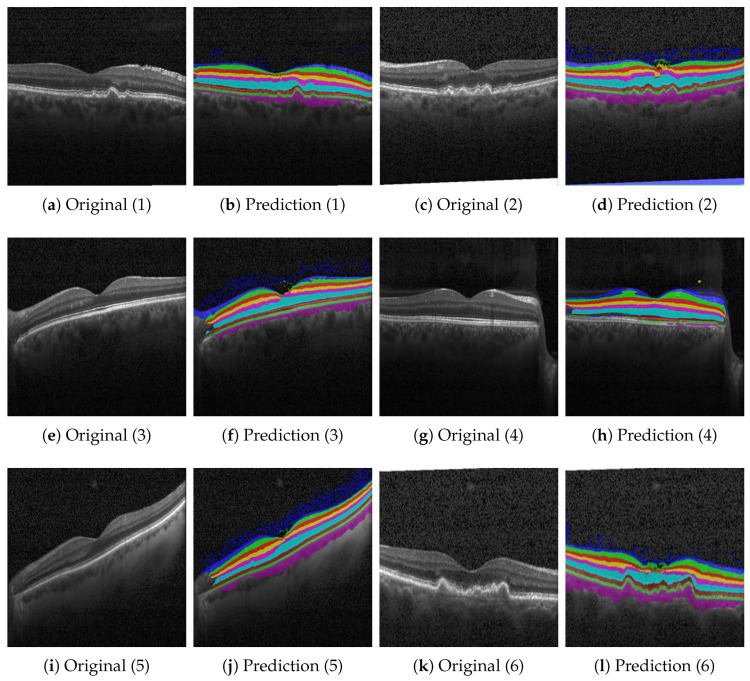
Qualitative results based on the Heidelberg OCT data set described in Section 2.1.4. For the Heidelberg OCT data set, no annotations are available. We consider the Heidelberg data set to be a particularly noisy data set. This is apparent when comparing Figure 5 with (**c**) and (**k**): General image noise and artifacts are observed.

**Figure 7:**
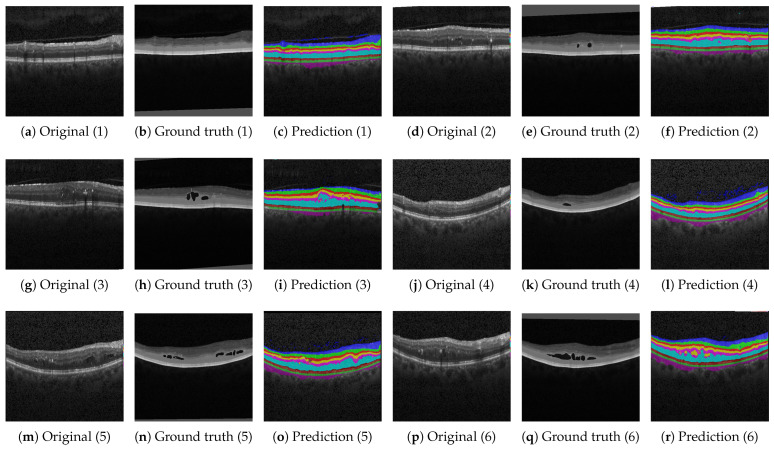
Qualitative results based on the Duke SD-OCT data set described in Section 2.1.2. The ground truth comprises fluid and non-fluid manual annotations of eight boundaries.

**Figure 8:**
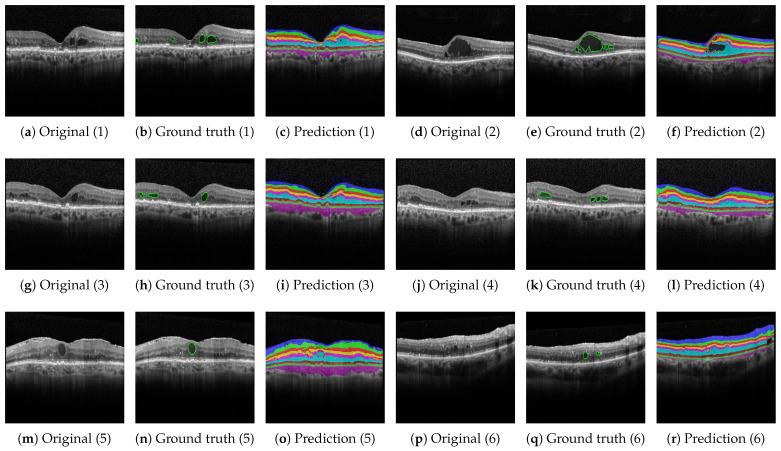
Qualitative results based on the UMN data set described in Section 2.1.3. The ground truth comprises the retinal fluid regions.

Following our quantitative results, Figure 5, Figure 6, Figure 7 and Figure 8 give an overview of our qualitative results in the form of the obtained segmentation visualizations, where the different detected retinal layers are mapped on top of the original imagery. Figure 5 shows our results for the peripapillary data set, given two different segmentations with the original imagery (a,d), their ground truth annotations (b,e), and their predicted annotations (c,f). Additionally, Table 6 shows selected segmentation results in terms of the Dice score given in Figure 5c,f, whereas the class-wise score distributions are illustrated. Overall, it is observable that the ground truth annotations align closely in terms of the different layers’ positions and their spatial separations. Only minor deviations, e.g., in the center of the annotations for the optic disc (c and f in comparison to b and e), are observable. This observation is also evident within the related results table, showing a segmentation score of 63.65% for Figure 5c. Besides the optic disc, GCL, IS/OS, and RPE are the more difficult retinal layers to segment.

On the contrary, Figure 6, Figure 7 and Figure 8 show results with the Heidelberg, Duke SD-OCT, and UMN data sets for six different segmentations with the original imagery, their ground truth annotations when available, and their predicted annotations. We consider the Duke SD-OCT, Heidelberg, and UMN data sets to be noisy data sets with different types and classes of retinal layers. This is especially apparent when comparing Figure 5 with Figure 6c,k: General image noise and artifacts can be observed. For our public data sets, Figure 6 (Heidelberg data set) depicts comparable visualizations of the obtained segmentations. Here, no noteworthy deviations from the observable retinal layers can be perceived. Finally, in Figure 7 (Duke SD-OCT data set) and Figure 8 (UMN data set), a comparison with the ground truth annotations of the evaluated data sets is made. While the Duke SD-OCT and UMN data sets do not provide any comprehensive annotations regarding the present retinal layers, we conclude that our trained model can be deployed instead to generate the said annotations with an improved quality for these data sets. Future contributions will have to investigate these automatically generated annotations further, for which subsequent annotations by medical doctors are planned.

In conclusion, we quantitatively evaluated our model with previously unseen data sets with different characteristics regarding the image quality as well as annotations. While we consider the Heidelberg data set to be a particularly noisy data set, it also shows different annotations with, in turn, different classes for retinal layers. Yet, qualitatively convincing results could be achieved over all additional data sets, despite fewer layers explicitly being annotated in their labeling processes. We therefore conclude that our model is able to adapt to previously unseen data, as evaluated on these data sets.

## 4. Conclusions and Outlook

To determine the progression of visual pathologies, optical-coherence-tomography-based retinal image is often utilized to investigate the influence of OCT biomarkers and their progression when visual pathologies such as age-related macula degeneration, cataracts, and glaucoma must be evaluated. While segmentations with improved quality might improve the treatment of patients with eye diseases, the realization of a (semi-)automated system could also provide the benefits of reduced treatment times and treatment costs, especially in areas with otherwise less or no access to medical aid in general.

As a couple of approaches to the task of OCT image segmentation of retinal layers already exist, fundamental approaches from machine learning and deep learning utilizing deep neural networks have shown the first promising results. In our contribution, we propose the application of a machine-learning-based multistage system of stacked multiscale encoders and decoders for the task of image segmentation. While this approach is able to leverage already-existing state-of-the-art deep neural networks in terms of their image segmentation performance, it also furthers their motivation by only combining models for the encoding and decoding stages that elevate the resulting image segmentation scores.

Given the evaluated peripapillary OCT data set, we obtained a final segmentation performance of up to 82.25±0.74% in terms of the Sørensen–Dice coefficient, outperforming the current best single-stage model by 1.55% with a score of 80.70±0.20%. We conclude that, by adding additional stages to the image segmentation process, improved segmentation scores can be reached. With our trained model, the Duke SD-OCT, Heidelberg, and UMN data sets were further investigated, showing that even noisy and previously unseen data sets’ retinal layers can be (semi-)automatically segmented to complement available annotations with as yet non-contained OCT biomarker segmentations.

Future, as well as more application-focused contributions, could be used to extend our approach by assessing not only additional segmentation imagery but also the following OCT image classification stage. This includes further OCT biomarkers such as disorganizations of the retinal inner layers (DRIL) between GCL, IPL, INL, and OPL [79] as well as hyper-reflective foci (HRF). To explore novel approaches to image segmentation and classification, different transformer-based models [57] with improved prediction scores, encompassing, i.a., data-efficient image transformers (DeiTs) as well as hybrid transformers [80,81,82], will be further assessed. Additionally, further strategies regarding data augmentation will have to be investigated. Finally, a (semi-)automated recommender system can be realized to enable the future on-site deployment of our realized system. 

## Figures and Tables

**Table 1:** Overview of our public data sets used for the qualitative assessment with their number of images, image resolution, and availability of annotations for segmentations (✓). There are no annotations available for the Heidelberg data set (-). For the annotation details see Section 2.1.2 and Section 2.1.3.

Data Set Name	Number of Images	Image Resolution	Annotations
Duke SD-OCT [14]	88	536×496	✓
UMN [54]	125	1024×496	✓
Heidelberg [55]	125	1024×992	-

**Table 2:** The data set color scheme for our data set with its retinal layer based segmentations, ranging from the retinal nerve fiber layer (RNFL) to the optic disc. Figure 2 gives an overview of our data set with a few selected samples and visualized retinal layers.

Layer	Color Scheme
Retinal nerve fiber layer (RNFL)	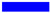
Ganglion cell layer (GCL)	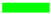
Inner plexiform layer (IPL)	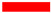
Inner nuclear layer (INL)	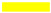
Outer plexiform layer (OPL)	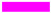
Outer nuclear layer (ONL)	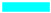
Inner/outer photoreceptor segment (IS/OS)	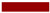
Retinal pigment epithelium (RPE)	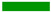
Choroid	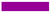
Optic disc	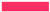

**Table 3:** Quantitative results for biomarker segmentation using nine different encoder–decoder stack combinations with one stack, evaluated on the peripapillary data set presented in Section 2.1. The Dice score was calculated using Equation (4). The best model is highlighted in bold. Table 4 shows our results using two stacks of our model.

Model ID	Encoder	Decoder	Dice Score [%]
0	EfficientNet B0	Modified Attention U-Net	77.65
1	EfficientNet B1	Modified Attention U-Net	78.21
2	EfficientNet B2	Modified Attention U-Net	78.67
3	EfficientNet B3	Modified Attention U-Net	79.81
4	EfficientNet B4	Modified Attention U-Net	76.82
5	EfficientNet B5	Modified Attention U-Net	77.02
6	ResNet34D	Modified Attention U-Net	80.14
7	ResNet50D	Modified Attention U-Net	80.24
8	SEResNeXt50-32x4D	Modified Attention U-Net	81.42

**Table 4:** The quantitative results of biomarker segmentation using 10 different encoder–decoder stack combinations with two stacks, evaluated on the peripapillary data set presented in Section 2.1. For the last three models, the three best models were evaluated by deploying their encoder as encoder 1 and encoder 2. The Dice score was calculated using Equation (4). The best model is highlighted in bold.

Model ID	Encoder 1	Encoder 2	Decoders 1 and 2	Dice Score [%]
0	EfficientNet B0	EfficientNet B1	Modified Attention U-Net	79.15±0.48
1	EfficientNet B2	EfficientNet B3	Modified Attention U-Net	80.55±0.28
2	EfficientNet B4	EfficientNet B5	Modified Attention U-Net	78.49±0.31
3	EfficientNet B0	ResNet34D	Modified Attention U-Net	80.67±0.26
4	EfficientNet B2	ResNet34D	Modified Attention U-Net	79.40±0.19
5	ResNet50D	ResNet34D	Modified Attention U-Net	80.88±0.28
6	SEResNeXt50-32x4D	ResNet34D	Modified Attention U-Net	82.25±0.74
7	ResNet34D	SEResNeXt50-32x4D	Modified Attention U-Net	80.82±0.41
8	SEResNeXt50-32x4D	EfficientNet B2	Modified Attention U-Net	79.39±0.27
9	SEResNeXt50-32x4D	EfficientNet B3	Modified Attention U-Net	78.71±0.34
10	ResNet34D	ResNet34D	Modified Attention U-Net	80.41±0.48
11	ResNet50D	ResNet50D	Modified Attention U-Net	80.17±0.19
12	SEResNeXt50-32x4D	SEResNeXt50-32x4D	Modified Attention U-Net	81.07±0.42

**Table 5:** Comparison of the current state-of-the-art method using a single-stage approach (first row) as well as our approaches with one stack with one encoder and one decoder (second row) and with two stacks of encoders and decoders (last row). The best model is highlighted in bold.

Model	Dice Score [%]
State-of-the-art model with a single stage [17]	80.70±0.20
One stack with one encoder and one decoder with attention pooling (SEResNeXt50-32x4D)	81.42±0.54
Two stacks of encoders and decoders with attention pooling (best model from Table 4, model ID 6: SEResNeXt50-32x4D and ResNet34D)	82.25±0.74

**Table 6:** Overview of individual, class-wise Dice score results following Figure 5. Overall there are 48 images.

Test Sample(s)	RNFL	GCL	IPL	INL	OPL	ONL	IS/OS	RPE	Choroid	Optic Disc	Overall Dice Score [%]
Figure 5c	85.05	75.21	79.12	81.73	84.13	83.63	51.89	67.84	92.91	63.65	76.52
Figure 5f	92.72	79.64	80.78	84.17	88.73	95.03	92.50	90.00	96.67	90.23	89.05
Overall	83.25	68.49	73.93	80.65	81.39	91.73	85.84	84.55	86.92	85.56	82.25

## Data Availability

We gratefully thank the authors of Li et al. [17] for providing us with their peripapillary OCT data set. The Duke SD-OCT [14], UMN [54], and Heidelberg [55] data sets are publically available.

## Abbreviations

The following abbreviations are used in this manuscript:

AMD	Age-related macula degeneration
DNN	Deep neural network
GCL	Ganglion cell layer
INL	Inner nuclear layer
IPL	Inner plexiform layer
IRF	Intraretinal fluid
IS/OS	Inner/outer photoreceptor segment
ONL	Outer nuclear layer
OPL	Outer plexiform layer
PED	Pigment epithelial detachment
RNFL	Retinal nerve fiber layer
RNN	Recurrent neural network
RPE	Retinal pigment epithelium
SRF	Subretinal fluid
